# Melatonin Reverses High-Temperature-Stress-Inhibited Photosynthesis in the Presence of Excess Sulfur by Modulating Ethylene Sensitivity in Mustard

**DOI:** 10.3390/plants12173160

**Published:** 2023-09-02

**Authors:** Noushina Iqbal, Zebus Sehar, Mehar Fatma, Sheen Khan, Ameena Fatima Alvi, Iqbal R. Mir, Asim Masood, Nafees A. Khan

**Affiliations:** 1 Department of Botany, Jamia Hamdard, New Delhi 110062, India; 2 Plant Physiology and Biochemistry Laboratory, Department of Botany, Aligarh Muslim University, Aligarh 202002, India

**Keywords:** melatonin, heat stress, ethylene, photosynthesis, S-assimilation, glutathione

## Abstract

Melatonin is a pleiotropic, nontoxic, regulatory biomolecule with various functions in abiotic stress tolerance. It reverses the adverse effect of heat stress on photosynthesis in plants and helps with sulfur (S) assimilation. Our research objective aimed to find the influence of melatonin, along with excess sulfur (2 mM SO_4_^2−^), in reversing heat stress’s impacts on the photosynthetic ability of the mustard (*Brassica juncea* L.) cultivar SS2, a cultivar with low ATP-sulfurylase activity and a low sulfate transport index (STI). Further, we aimed to substantiate that the effect was a result of ethylene modulation. Melatonin in the presence of excess-S (S) increased S-assimilation and the STI by increasing the ATP-sulfurylase (ATP-S) and serine acetyltransferase (SAT) activity of SS2, and it enhanced the content of cysteine (Cys) and methionine (Met). Under heat stress, melatonin increased S-assimilation and diverted Cys towards the synthesis of more reduced glutathione (GSH), utilizing excess-S at the expense of less methionine and ethylene and resulting in plants’ reduced sensitivity to stress ethylene. The treatment with melatonin plus excess-S increased antioxidant enzyme activity, photosynthetic-S use efficiency (p-SUE), Rubisco activity, photosynthesis, and growth under heat stress. Further, plants receiving melatonin and excess-S in the presence of norbornadiene (NBD; an ethylene action inhibitor) under heat stress showed an inhibited STI and lower photosynthesis and growth. This suggested that ethylene was involved in the melatonin-mediated heat stress reversal effects on photosynthesis in plants. The interaction mechanism between melatonin and ethylene is still elusive. This study provides avenues to explore the melatonin–ethylene-S interaction for heat stress tolerance in plants.

## 1. Introduction

Mustard (*Brassica juncea* L.) is the third most important oilseed crop, after soybean and oil palm, in the world. Many species of mustard are mainly grown in the world’s temperate regions. In India, it is cultivated on over 7 million hectares in the northwestern states when irrigated and rainfed [1]. Mustard is a winter crop that exhibits an efficient photosynthetic response at 15–20 °C and is affected by high temperatures [2]. Indeed, a sudden increase in temperature causes severe consequences for its growth and plant productivity via the overproduction of reactive oxygen species (ROS) [3,4]. The reduction in chlorophyll levels, damage to the thylakoid membrane, decreased photosystem II, and distortion of the oxygen-evolving complex are symptoms of the cellular oxidative damage caused by ROS [5,6,7]. The decrease in photosynthetic-S use efficiency, photosynthetic-N- use efficiency, which results in decreased Rubisco activity and photosynthetic capacity, is one of the additional impacts of high temperatures [7]. According to [8], the high S requirement of mustard is a key factor in reducing oxidative stress. Protein, glucosinolate, sulfolipids, and the thioredoxin system are some of the major metabolic compounds that contain S. These compounds have the potential to harmonize biochemical and physiological processes to accelerate plants’ ability to resist abiotic stress [9]. In order to control the degree of oxidative damage, plants under abiotic stress conditions upregulate the antioxidant system to increase GSH production via enhanced Cys synthesis resulting from the increased activity of S-assimilatory enzymes (ATP-S and serine acetyltreansferase; SAT) and S-assimilation [10].

Melatonin (N-acetyl-5-methoxy tryptamine) is a growth regulator, and it possesses a natural antioxidant property [11,12]. In response to an abiotic stress factor, such as temperature stress, it is crucial for plants’ survival [12,13]. It protects the photosynthetic efficiency of plants under diverse unfavorable conditions [11,13,14].

Ethylene is a gaseous signaling molecule involved in plants’ tolerance for high temperature stress [7,15]. Its relative proportion in plant tissues acts as an indicator of prevailing stress or plants’ enhanced metabolic activities. In our earlier study [7], we found that increased ethylene levels under heat stress caused the inhibition of photosynthetic potential and yield. However, the crosstalk between melatonin and ethylene for high temperature stress tolerance in the presence of excess-S is elusive, and it requires thorough study.

Sulfur is an essential nutrient element linked with ethylene synthesis via Cys formation [8]. In order to tolerate abiotic stress, ethylene and S-assimilation studies have been published [15], but information on the interaction between melatonin and S-assimilation is not clear. Moreover, the coordination of melatonin, S-assimilation, and ethylene sensitivity in the protection of photosynthesis and high temperature stress tolerance is completely unknown.

This study focused on obtaining detailed information on how melatonin regulates ethylene sensitivity and the photosynthetic performance of the low ATP-S activity SS2 cultivar of mustard in the presence of excess-S in order to mitigate the negative impacts of high temperature stress. In the earlier research, the SS2 cultivar showed low ATP-S activity and less salt tolerance [10]. Therefore, we selected this cultivar to study whether, in the presence of excess-S and a promoter (melatonin) that enhances S uptake and assimilation, the photosynthetic potential of this cultivar is increased, together with its stress tolerance ability. Further, due to the link between S and ethylene, it becomes relevant to discuss whether this link has any effect on the reduction in stress ethylene and, thus, ethylene sensitivity.

## 2. Results

### 2.1. Melatonin with Excess-S Reduces High-Temperature-Associated Oxidative Stress

High-temperature-induced oxidative stress was responsible for cellular damage in plants, which was estimated via monitoring H_2_O_2_ content, and damage to the membrane was estimated via measuring thiobarbituric acid reactive substances (TBARS) content. High temperature stress significantly enhanced H_2_O_2_ and TBARS content by 71.5% and 85%, respectively, relative to the control plants. The supplementation of melatonin without stress decreased the H_2_O_2_ content by 47.0% and TBARS by 54.0% relative to the control. In contrast, SO_4_^2−^, when applied alone under no stress conditions, was inhibitory and, rather, increased H_2_O_2_ and TBARS content compared to the control. Exogenously applied melatonin and SO_4_^2−^ were efficient in reducing high-temperature-induced oxidative stress, lowering H_2_O_2_ content by 44.6% and 34.3%, respectively, and TBARS content by 50% and 40%, compared to high-temperature-treated plants (Table 1). The application of both melatonin and SO_4_^2−^ decreased oxidative stress to the greatest extent via lowering H_2_O_2_ and TBARS levels, implying improved S utilization with melatonin under heat stress.

### 2.2. Melatonin and Excess-S Accelerate Antioxidative Enzymes’ Activity under High Temperature Stress

The activity of antioxidant enzymes increased prominently in response to high temperature stress. In contrast to the control, the high temperature enhanced the activities of the antioxidant enzymes SOD by 85.7%, APX by 57.6%, and GR by 50%, respectively. The treatment of individual melatonin and SO_4_^2−^ increased SOD activity by 118.4% and 106.5%, APX activity by 120% and 33%, and GR activity by 142.8% and 100%, respectively, compared to the no-stress control plants. Furthermore, when exposed to high temperature stress, the exogenous supplementation of melatonin and SO_4_^2−^ resulted in a significant enhancement in ROS-scavenging enzyme activities with respective increases in SOD of 52.8% and 42.4%, in APX by 156.6% and 121.6%, and in GR by 109.5% and 85.7%, relative to the plants exposed to the high-temperature condition. However, the plants exposed to both melatonin and SO_4_^2−^ under high temperature stress exhibited the maximum significant increases in SOD activity by 81%, in APX by 193.7%, and in GR by 147.6%, compared to the temperature-stressed plants (Table 1).

### 2.3. Melatonin and Excess-S Effect on S-Assimilation and GSH Content under High Temperature Stress

High temperature stress increased S assimilation significantly through an increase in ATP-S and SAT activity and Cys, Met and reduced glutathione (GSH) content increased relative to the plants grown under control conditions. The application of melatonin alone also stimulated increases in Cys content by 46.5%, in Met content by 50.3%, in ATP-S activity by 49.7%, and in SAT activity by 91.3% compared to the plants grown under control conditions. Under high temperature stress, the treatment with melatonin/SO_4_^2−^ exhibited significant increases in Cys content by 39.6% and 13.9%, in ATP-S activity by 27.7% and 20.8%, and in SAT activity by 41.4% and 32.0%, while the Met content decreased by 44.2% and 54.5%, respectively, compared to the high-temperature-stress-exposed plants. However, the Met content was higher compared to the control. In addition to this, the plants treated with melatonin in conjunction with SO_4_^2−^ under high temperature stress exhibited the maximum increases in ATP-S activity by 58.4%, in SAT activity by 117.3%, and in Cys content by 60.9%, while Met decreased by 36.9%, compared to high-temperature-stress-grown plants (Table 2).

High temperature stress significantly enhanced GSH content by 17.6% in comparison to the non-treated control plants. The supplementation of melatonin and SO_4_^2−^ individually enhanced the GSH content by 41.4% and 19.7%, respectively, compared to the control plants. Moreover, melatonin/SO_4_^2−^ increased GSH content by 27.5% and 11%, respectively, in the presence of a high temperature, compared to the plants grown under temperature stress conditions. However, applied melatonin in the presence of SO_4_^2−^ and high temperature stress resulted in 58.5% greater GSH synthesis and, consequently, a reduction in oxidative stress with lower H_2_O_2_ and TBARS content (Table 2).

### 2.4. Effect of Melatonin and Excess-S on Root and Leaf Sulfate Content and the Sulfur Transport Index under High Temperature Stress

Root and leaf sulfate content decreased with an increase in temperature but increased the STI by 7.9%, relative to the control. Melatonin individually increased root and leaf sulfate content. In contrast, excess-S decreased root sulfate content, while leaf sulfate was significantly equal to the control. Excess-S alone reduced STI, while melatonin showed an increase in the STI by 25.2% compared to the plants grown under the control conditions. Under heat stress, melatonin/ SO_4_^2−^ triggered the content of root and leaf sulfate and also significantly increased the STI by 13% and 7.5%, respectively, compared to the high-temperature-stress-exposed plants. Moreover, the combined treatment using melatonin with SO_4_^2−^ under high temperature stress showed the maximum increase in the above-stated parameters compared to the plants under high temperature stress (Table 3).

### 2.5. Impact of Melatonin and Excess-S on 1-Aminocyclopropane Carboxylic Acid Synthase (ACS) Activity and Ethylene Evolution under High Temperature Stress

**Figure 1:**
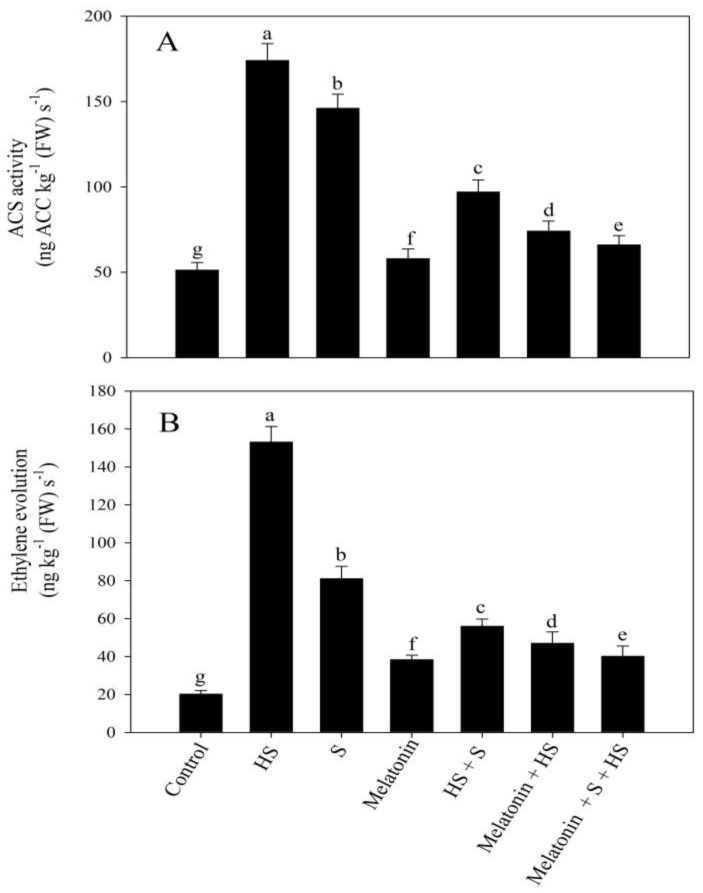
Activity of 1-aminocyclopropane-1-carboxylic acid synthase (ACS) (**A**) and ethylene evolution (**B**) of mustard (*Brassica juncea* L. cv. SS2) at 30 d after sowing. Plants were foliar treated with 100 of µM melatonin and/or 2 mM of SO_4_^2−^ (S) and grown with/without high temperature stress (HS; 40 °C for 6 h every day for 15 days). Data are presented as treatment means ± SEs (n = 4). Data followed by the same letter are not significantly different from the LSD test at *p* < 0.05. FW, fresh weight.

High temperature stress stimulated ACS activity and the production of ethylene by 240% and 657.4%, respectively, in comparison to the control. Individually, both melatonin and SO_4_^2−^ increased ACS activity, by 13.3% and 185%, and ethylene production, by 89.6% and 300%, respectively, compared to the no-stress control. Melatonin and SO_4_^2−^ under high temperature stress significantly decreased ACS activity, by 57.5% and 44.3%, and ethylene production, by 70% and 63.4%, respectively, in comparison to the high-temperature-stress-exposed plants (Figure 1). Finally, under high temperature stress, the treatment using melatonin and SO_4_^2−^ caused the maximum decreases in ACS activity and ethylene production by 62% and 74%, respectively, in comparison to the plants exposed to high temperature stress (Figure 1).

### 2.6. Effect of Melatonin and Excess-S on Rubisco Activity and Photosynthetic Sulfur Use Efficiency (p-SUE) under High Temperature Stress

**Figure 2:**
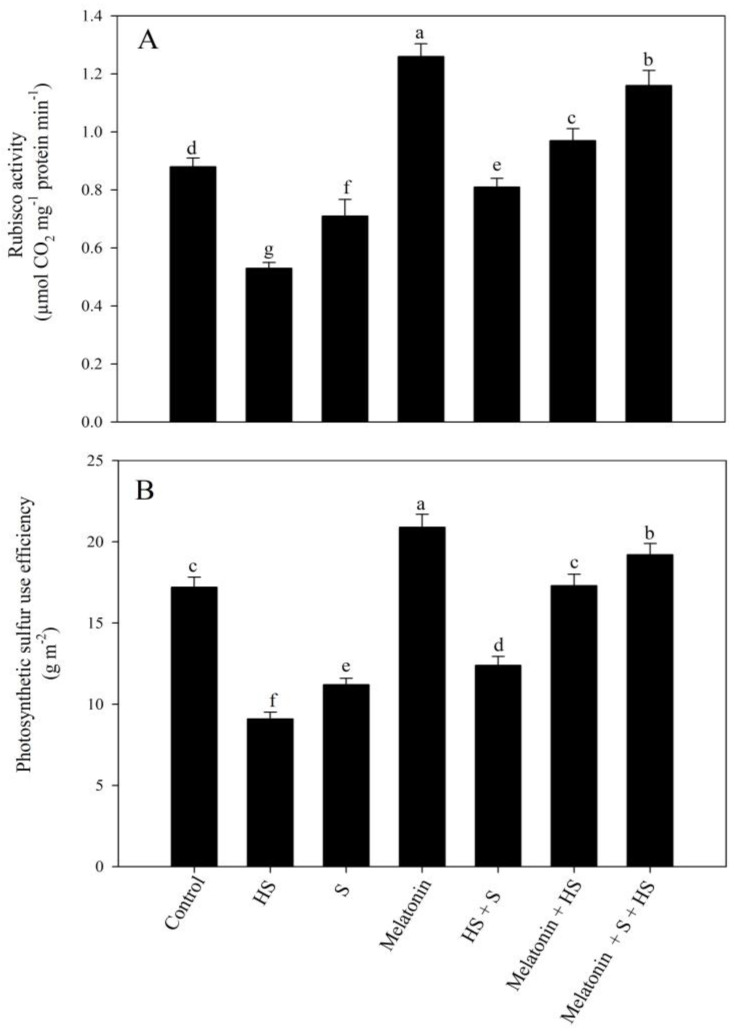
Rubisco activity (**A**) and photosynthetic sulfur use efficiency (p-SUE) (**B**) of mustard (*Brassica juncea* L. cv. SS2) at 30 d after sowing. Plants were foliar treated with 100 µM of melatonin and/or 2 mM of SO_4_^2−^ (S) and grown with/without high temperature stress (HS; 40 °C for 6 h every day for 15 days). Data are presented as treatment means ± SEs (n = 4). Data followed by the same letter are not significantly different from the LSD test at *p* < 0.05.

In comparison to the control plants, high temperature stress reduced Rubisco activity and photosynthetic-sulfur use efficiency (p-SUE) by 39.8% and 47.1%, respectively. The individual application of melatonin resulted in significant increases in Rubisco activity by 43.2% and in p-SUE by 21.5%, while excess-S decreased Rubisco activity by 19.3% and p-SUE by 34.8%, in comparison to the control. Under temperature stress, the treatment using melatonin and SO_4_^2−^ showed enhancements in Rubisco activity by 83% and 52.8% and in p-SUE by 90% and 36.3%, respectively, in comparison to the plants exposed to high-temperature-stress conditions. Meanwhile, under high-temperature-stress conditions, the plants receiving melatonin and SO_4_^2−^ exhibited improved Rubisco activity and p-SUE by 119% and 111%, respectively, compared to the plants exposed to high temperature stress (Figure 2).

### 2.7. Effect of Melatonin and Excess-S on Gas Exchange and Growth Attributes under High Temperature Stress

High temperature stress decreased net photosynthesis (*Pn*), stomatal conductance (*Gs*), and intercellular CO_2_ concentration (*Ci*) by 37.8%, 28.4%, and 26.9%, respectively, relative to the control plants. Meanwhile, the individual application of melatonin resulted in higher values of *Pn, Gs,* and *Ci* relative to the control plants. On the contrary, excess-S via 2 mM of SO_4_^2−^ reduced all the studied photosynthetic traits compared to the control plants. However, both melatonin/SO_4_^2−^ in the presence of high temperature stress demonstrated increments in *Pn, Gs,* and *Ci* in comparison to the plants exposed to high-temperature-stress conditions. Melatonin plus S reversed the effects of high temperature stress and improved the photosynthetic characteristics compared to the control and heat-stressed plants. The application of melatonin in the presence of SO_4_^2−^ under high-temperature-stress conditions resulted in a significant and maximum increase in *Pn*, *Gs,* and *Ci* compared to the high-temperature-exposed plants (Table 4).

**Figure 3:**
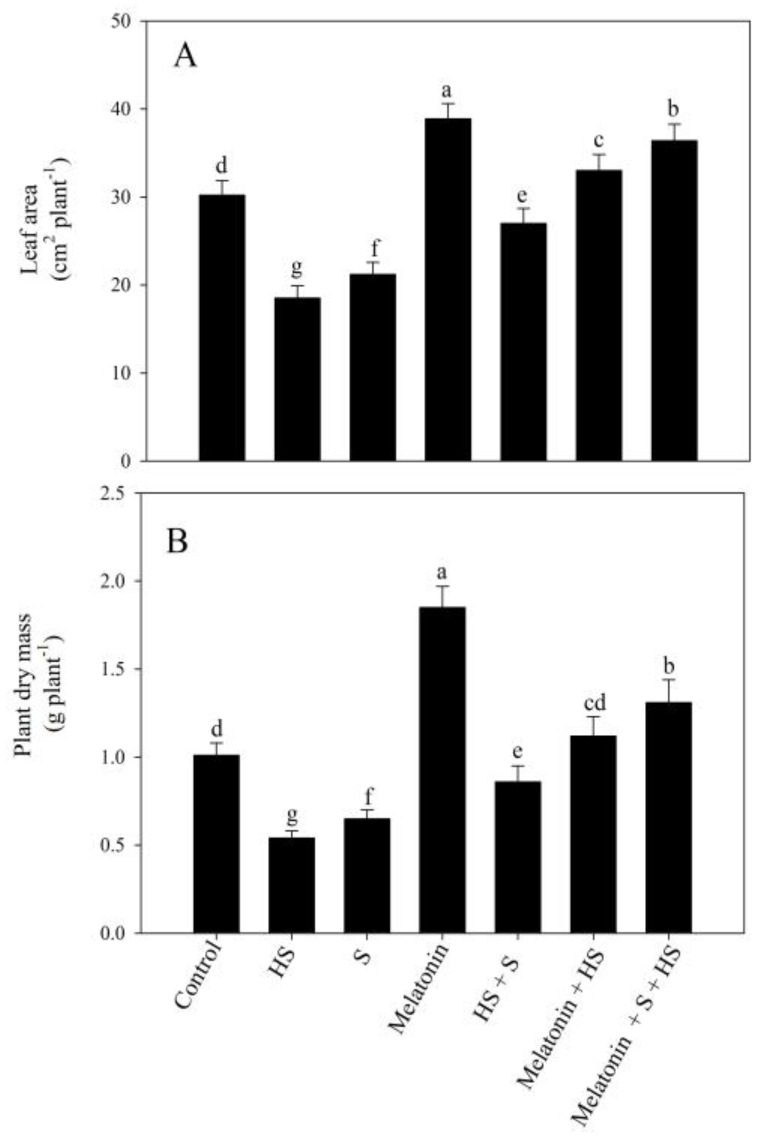
Leaf area (**A**) and plant dry mass (**B**) of mustard (*Brassica juncea* L.) cv. SS2 at 30 d after sowing. Plants were foliar treated with 100 µM of melatonin and/or 2 mM of SO_4_^2−^ (S) and grown with/without high temperature stress (HS; 40 °C for 6 h every day for 15 days). Data are presented as treatment means ± SEs (n = 4). Data followed by the same letter are not significantly different from the LSD test at *p* < 0.05.

In particular, exposure to the high temperature severely hampered plant growth characteristics, determined as leaf area and plant dry mass, by 38.7% and 46.5%, respectively, compared to the control plants. However, leaf area and plant dry mass were enhanced via the individual application of melatonin, but SO_4_^2−^ application decreased these under the no-stress condition. On the contrary, under high-temperature conditions, the treatment with melatonin/SO_4_^2−^ exhibited a significant increase in leaf area and plant dry mass in comparison to the plants exposed to high temperature stress conditions. Moreover, the melatonin and SO_4_^2−^ application in conjunction with high temperature stress improved leaf area and plant dry mass by approximately 2 and 2.4 times, respectively, compared to the high-temperature-exposed plants (Figure 3).

### 2.8. Application of NBD Suppresses Melatonin Effects under High Temperature Stress

**Figure 4:**
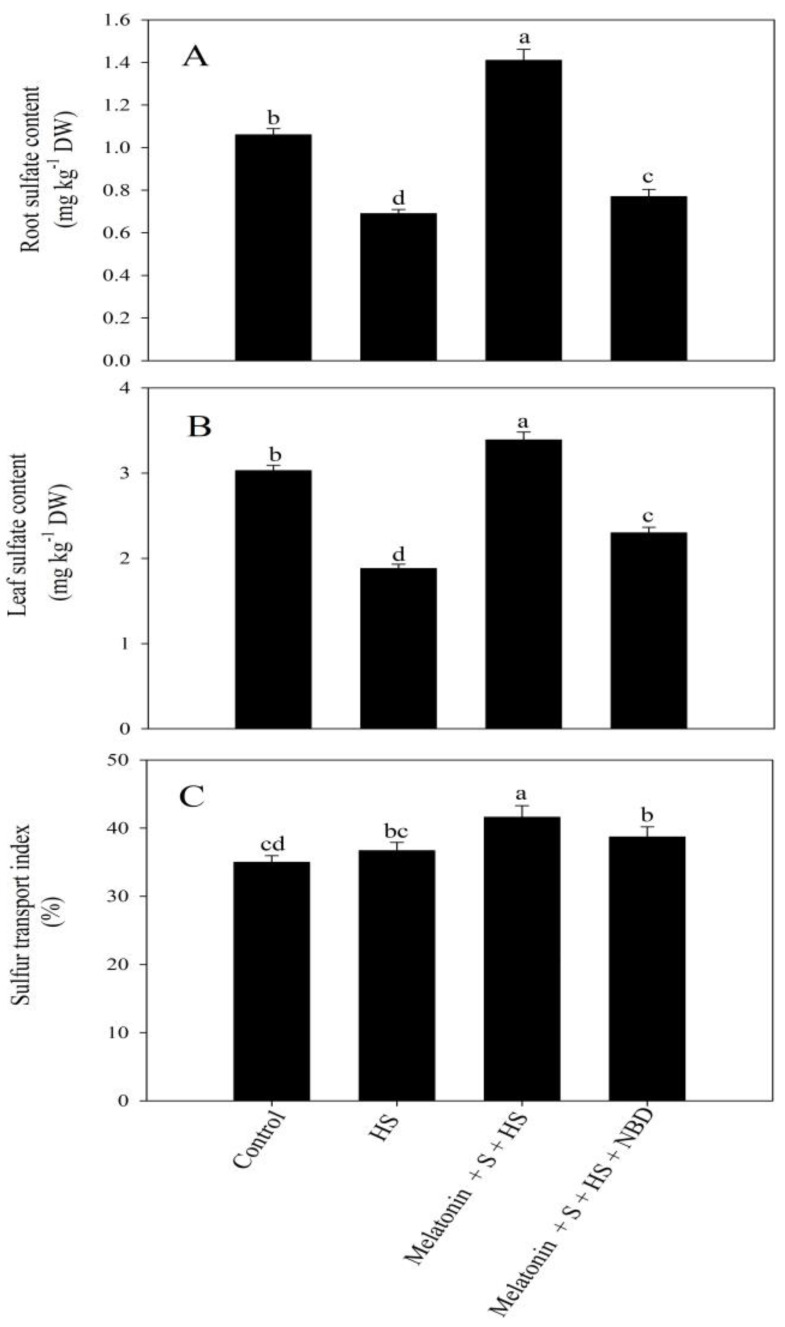
Root sulfate content (**A**), leaf sulfate content (**B**), and sulfur transport index (**C**) of mustard (*Brassica juncea* L. cv. SS2) at 30 d after sowing. Plants were foliar treated with 100 µM of melatonin and/or 2 mM of SO_4_^2−^ (S) and grown with/without high temperature stress (HS; 40 °C for 6 h every day for 15 days). Data are presented as treatment means ± SEs (n = 4). Data followed by the same letter are not significantly different from the LSD test at *p* < 0.05.

High temperature stress decreased root sulfate content (RSC) and leaf sulfate content (SSC) by 35% and 38%, respectively, but increased the sulfur transport index (STI) by 4.9% in comparison to the control. The application of melatonin with SO_4_^2−^ under high temperature stress increased these characteristics maximally in comparison to the heat-exposed plants. However, the supplementation of norbornadiene (NBD) in the presence of melatonin and SO_4_^2−^ under high temperature stress decreased RSC, SSC, and the STI by 45.4%, 32.2%, and 7%, respectively, compared to the plants receiving melatonin and SO_4_^2−^ under high-temperature-stress conditions (Figure 4).

**Figure 5:**
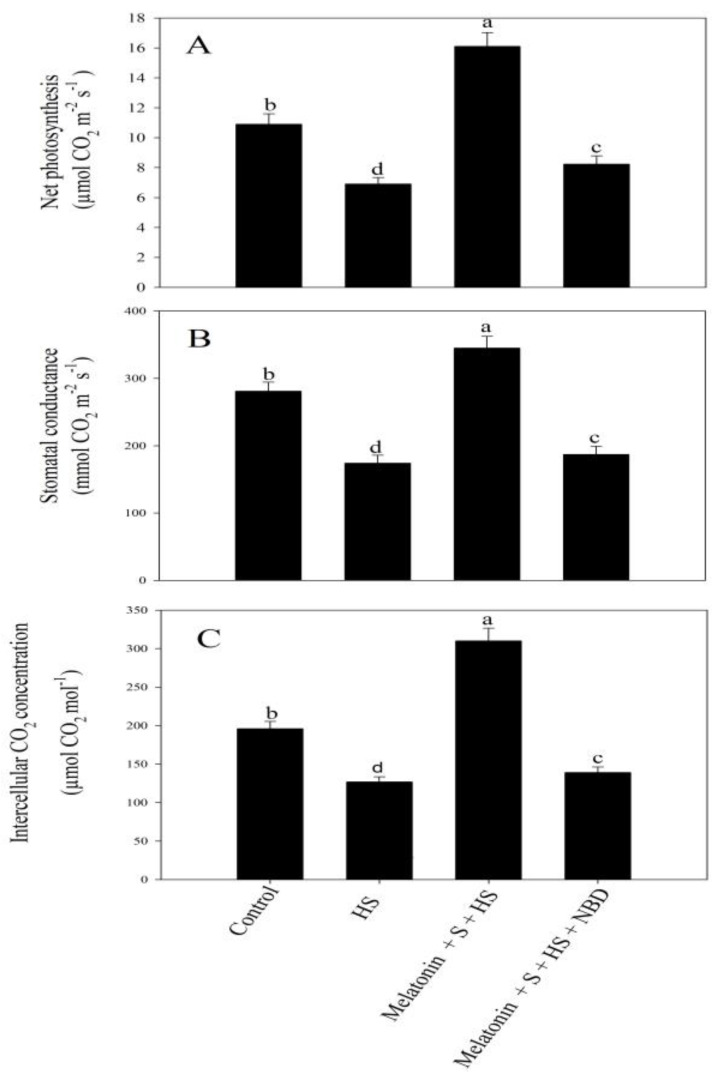
Net photosynthesis (Pn) (**A**), stomatal conductance (Gs) (**B**), and intercellular CO_2_ concentration (Ci) (**C**) of mustard (*Brassica juncea* L. cv. SS2) at 30 d after sowing. Plants were grown with/without high temperature stress (HS; 40 °C for 6 h every day for 15 days) and were foliar treated with 100 µM of melatonin and 2 mM of SO_4_^2−^ (S) with/without 100 µM norbornadiene (NBD). Data are presented as treatment means ± SEs (n = 4). Data followed by the same letter are not significantly different from the LSD test at *p* < 0.05.

**Figure 6:**
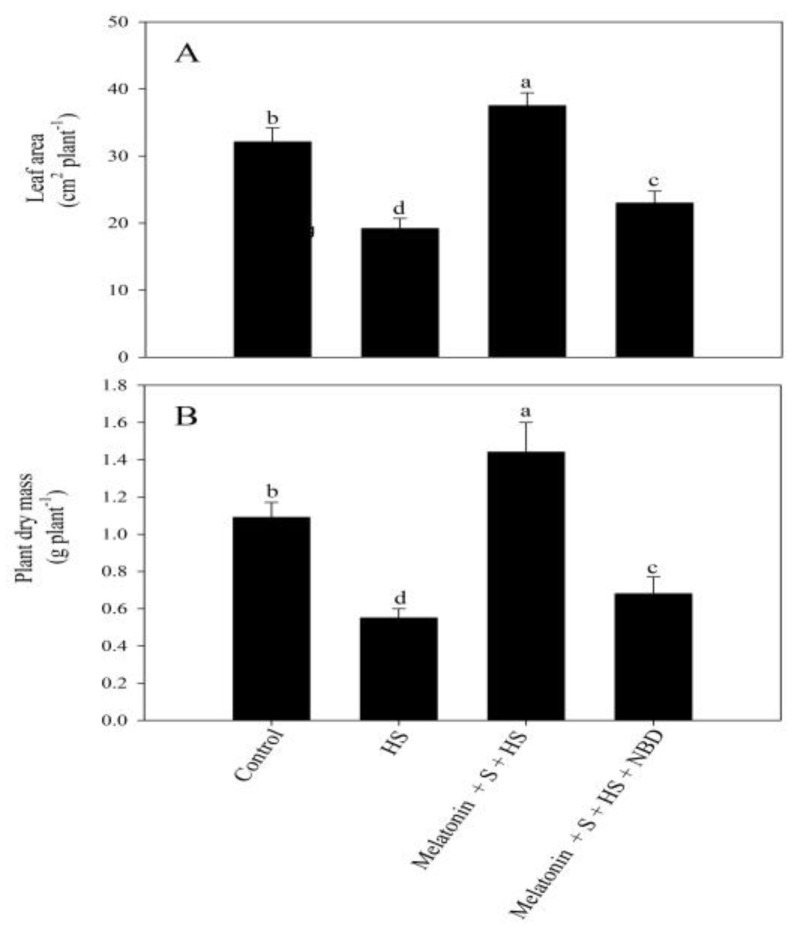
Leaf area (**A**) and plant dry mass (**B**) of mustard (*Brassica juncea* L. cv. SS2) at 30 d after sowing. Plants were grown with/without high temperature stress (HS; 40 °C for 6 h every day for 15 days). Heat-stressed plants were foliar treated with 100 µM of melatonin and 2 mM of SO_4_^2−^ (S) with/without 100 µM norbornadiene (NBD). Data are presented as treatment means ± SEs (n = 4). Data followed by the same letter are not significantly different from the LSD test at *p* < 0.05.

A similar effect of NBD was observed on photosynthetic and growth characteristics through which, even in the presence of melatonin and excess SO_4_^2−^, no improvement in photosynthesis (Figure 5) or growth (Figure 6) was observed in heat-stressed plants.

This suggests that melatonin in the presence of excess-S regulates ethylene sensitivity, enhancing S-assimilation and antioxidative metabolism to reduce oxidative stress, which increases photosynthetic and growth characteristics even in a low ATP-S activity cultivar. NBD’s inhibiting ethylene action, thus, resulted in inhibited root and shoot sulfate content, photosynthesis, and growth. The use of NBD showed ethylene’s involvement in regulating melatonin and sulfate-mediated responses under heat stress.

### 2.9. Principal Component Analysis

**Figure 7:**
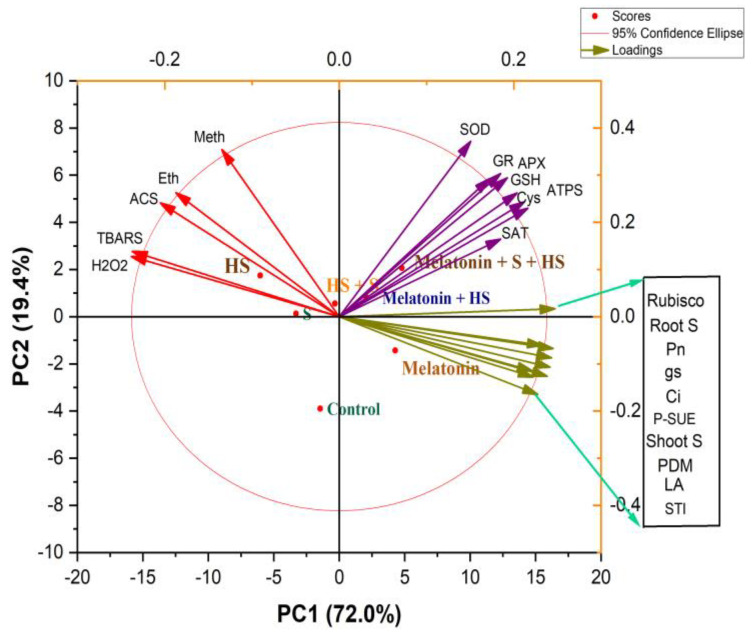
Principal component analysis (PCA) biplot for growth and physio–biochemical traits of *Brassica juncea* plants. The treatments included control, heat stress (HS), sulfur (S), melatonin, HS + S, melatonin + HS, and melatonin + S + HS. The variables included methionine (Meth), ethylene (Eth), 1-aminocyclopropane carboxylic acid synthase (ACS), thiobarbituric-acid-reactive substances (TBARS), hydrogen peroxide (H_2_O_2_), superoxide dismutase (SOD), glutathione reductase (GR), ascorbate peroxidase (APX), reduced glutathione (GSH), cysteine (Cys), ATP-sulfurylase (ATP-S), serine acetyltransferase (SAT), Rubisco activity, root S, leaf S, sulfate transport index (STI), net photosynthesis (Pn), stomatal conductance (gs), intercellular CO_2_ concentration (Ci), photosynthetic-S use efficiency (p-SUE), plant dry mass (PDM), and leaf area (LA).

Figure 7 depicts the principal component analysis (PCA) scores used to assess the effects of melatonin and S on *B. juncea* plants during heat stress. PC1 and PC2 accounted for 91.4% of the dataset’s total variation. PC1 supplied 72.0% of the total variance, whereas PC2 contributed 19.4%. The first two main components successfully disseminated all of the treatments (Figure 7). The observed parameters in the PCA biplot were classified into three major groups. The oxidative stress biomarkers (H_2_O_2_ and TBARS content), together with stress ethylene content, methionine content, and ACS activity, were all distributed along with the treatment using high-temperature stress. The parameters of growth, photosynthesis, and SUE were similar to those of the melatonin treatment without stress. On the other hand, those of antioxidants (SOD, APX, GR, GSH), Cys content, and ATP-S and SAT activity were similar to those of the combined treatment using melatonin and S in the presence of high temperature stress (Figure 7). The oxidative stress biomarkers and ethylene biosynthesis showed a negative correlation with the plant growth and photosynthesis parameters. From the biplot, it is clear that the antioxidants and components of S-assimilation were more concentrated in the combined heat-plus-melatonin-and-S treatment, demonstrating that they were maximally increased via this treatment and that they lay between oxidative stress and plant growth and photosynthesis, suggesting their role in combating heat stress. Therefore, the correlation biplot depicts a close association between S and melatonin in the heat stress acclimation of *B. juncea* plants (Figure 7).

### 2.10. Pearson Correlation

**Figure 8:**
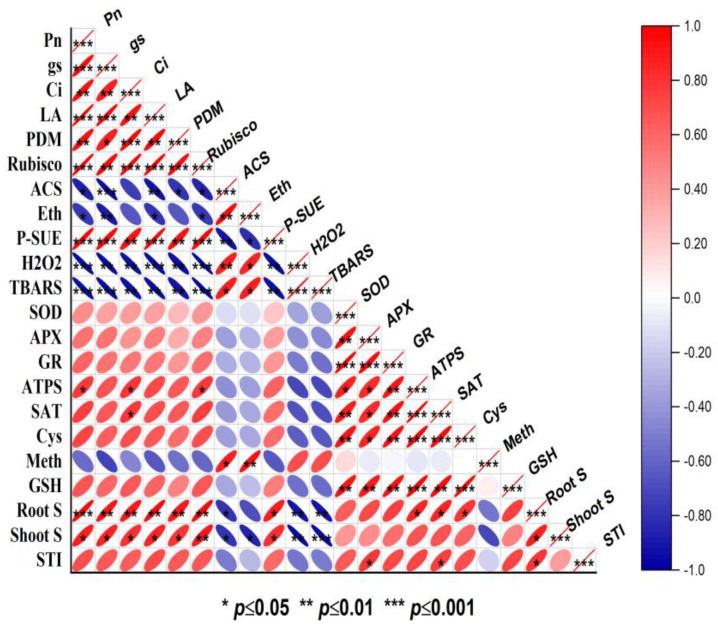
Pearson correlation heatmap showing the relationship among different observed variables for *Brassica juncea* plants. The treatments included control, heat stress (HS), sulfur (S), melatonin, HS + S, melatonin + HS, and melatonin + S + HS. The variables included methionine (Meth), ethylene (Eth), 1-aminocyclopropane carboxylic acid synthase (ACS), thiobarbituric acid reactive substances (TBARS), hydrogen peroxide (H_2_O_2_), superoxide dismutase (SOD), glutathione reductase (GR), ascorbate peroxidase (APX), reduced glutathione (GSH), cysteine (Cys), ATP-sulfurylase (ATP-S), serine acetyltransferase (SAT), Rubisco activity, root S, leaf S, sulfate transport index (STI), net photosynthesis (Pn), stomatal conductance (gs), intercellular CO_2_ concentration (Ci), photosynthetic-S use efficiency (p-SUE), plant dry mass (PDM), and leaf area (LA).

To investigate the link between the investigated characteristics, a Pearson correlation heatmap was created (Figure 8). A substantial (*p* ≤ 0.05; *p* ≤ 0.01 and *p* ≤ 0.001) positive association was found between the observed growth, photosynthesis, antioxidant, and S-metabolism properties. Heat-stress-induced oxidative stress showed a negative correlation with plant growth and photosynthetic attributes. Ethylene production and ACS activity, on the other hand, showed a strong correlation with H_2_O_2_, TBARS, and Met content, showing the stress-specific production of ethylene. The antioxidant enzymes (SOD, APX, and GR) and S-assimilation components (ATP-S, SAT, GR, Cys, and GSH and S content) were inversely linked with H_2_O_2_ and TBARS content. Furthermore, the STI showed a positive correlation with PDM, LA, *Pn, Gs, Ci,* and Rubisco activity, indicating that S-assimilation has a favorable function in enhancing growth and photosynthesis under heat stress (Figure 8).

## 3. Discussion

In plants, high temperature stress induces excessive ROS generation and has negative consequences for plant development [7]. To cope with these adversities, plants are supplemented with various growth regulators. In this study, we investigated the potential of melatonin with excess-S to control ethylene levels, photosynthesis, and growth in mustard under heat stress. The results were confirmed using the ethylene action inhibitor NBD.

### 3.1. Melatonin along with Excess-S Reverses the Heat-Stress-Associated Negative Impact on Plants’ Photosynthetic Attributes and Growth

A reduction in photosynthetic efficiency is one of the unavoidable consequences of heat stress. A reduced photosynthetic rate reduces a plant’s physiological and growth characteristics. High temperature stress alters the characteristics of the photosynthetic apparatus and Rubisco activity [7]. It was found that the combined treatment of melatonin and S had the highest photosynthesis efficiency and other relevant photosynthetic parameters. Under heat stress, melatonin enhanced *Pn, gs, Ci*, and p-SUE the most. Similar results have been reported for tomatoes, wherein the melatonin and S combination alleviated lanthanum (La)-toxicity [16], suggesting that the combination is useful for plants subjected to abiotic stress. The application of melatonin and S resulted in the enhanced activity of antioxidants and reduced photosynthetic damage in terms of chlorophyll protection. This study is unique because it mainly focuses on the outcome of this combination for the lower-ATPS-activity mustard cultivar. Enhancing the photosynthesis during heat stress of the low-ATP-S cultivar is more challenging because of its lower STI and lower GSH content. The strategy of enhancing its S-assimilation with melatonin supplementation helped to increase heat tolerance in a cultivar with low S-assimilation.

Through its effect on root development, melatonin indirectly regulates nutrient absorption [17]. Similarly, in this study, melatonin increased the p-SUE and S content in leaves, resulting in enhanced Rubisco activity and photosynthesis, particularly affecting the availability of S under heat stress. On the contrary, we observed reduced net photosynthesis and p-SUE with a concomitant reduction in growth in the plants treated with excess-S only. Studies have shown that optimum concentrations, as well as an absorbable S form, are a must in the soil for proper uptake and assimilation [18]. Excessive S can reduce photosynthesis and growth in mustard due to stress ethylene evolution, as the S-assimilation pathway is directed to ethylene synthesis via Met and not to GSH formation. However, during salt stress, increased demand for S leads to the increased uptake and assimilation needed to produce optimum ethylene formation, leading to greater GSH synthesis and promoting photosynthesis and growth [8]. Further, melatonin helped increase the STI and GSH content with a reduction in stress ethylene to protect photosynthesis under heat stress. It was found to increase S-uptake and assimilation and reduce oxidative stress caused by heat through GSH formation and enhanced antioxidative enzyme activity. This led to the protection of chlorophyll and an increase in net photosynthesis. Sulfur assimilation increases chlorophyll content and Rubisco activity, which increases CO_2_ assimilation in plants and, thus, enhances photosynthesis and growth. 

### 3.2. Melatonin Alone and with Excess-S Regulates S-Assimilation for Heat Tolerance

Melatonin with excess S, in this study, helped increase S-assimilation under heat stress. This utilization of S occurred because the S assimilation pathway is a demand-driven pathway that regulates S based on its requirement by plants [19,20,21,22]. The S-assimilation pathway is typically repressed when there is an adequate external sulfate supply, and it is depressed via sulfate limitation, i.e., when plants require S [23,24]. Plants require sufficient S for proper growth, but excess-S induces oxidative stress and growth inhibition [18]. In this study, excess-S caused oxidative stress, while S-assimilation increased as there was a requirement for S compounds, such as GSH, under heat stress. Moreover, melatonin further enhanced S-assimilation, GSH synthesis, and stress ethylene reduction to alleviate the heat-stress-induced negative effect on photosynthesis. A similar study has been conducted on La stress; it showed that the combined melatonin and S application was more effective in decreasing ROS accumulation in La-treated seedlings through higher S uptake, which enhanced ROS detoxification via antioxidative enzymes’ up-regulation [25], fulfilling the thiols’ demand to balance the production of ROS [16,26].

Under high temperature stress, plants need these S-compounds; therefore, excess-S was not harmful to the plants under heat stress, unlike the plants under normal conditions. Melatonin under heat stress also increased S-assimilation to promote a reduction in ROS through an increase in GSH content. It was observed in our study that SAT and ATP-S activity, and consequently Cys and GSH content, increased with melatonin and excess-S, suggesting a demand-driven increase in S. Hasan et al. [27] reported the involvement of melatonin in inducing S-transport and its assimilation and metabolism to maintain redox homeostasis, which helped with Cd tolerance. A sulfur foliar treatment resulted in heat stress tolerance in tomatoes and improved their physiological responses and growth through an increase in ascorbate and GSH content that subsequently lowered H_2_O_2_, electrolyte leakage, and MDA [28]. We observed a decrease in Met content via S and melatonin application, suggesting that Met is diverted toward ethylene formation. This diversion occurs because S-assimilation leads to Met synthesis from Cys and via S-adenosyl methionine (SAM) to ethylene, establishing the relationship of S and Met with ethylene [29]. Cysteine is a common precursor to both Met and GSH, and there was a higher increase in GSH and correspondingly lower Met and ethylene. A previous study reported that melatonin results in ethylene suppression [30] for ozone tolerance. However, the interaction between melatonin, excess-S, and ethylene for heat stress tolerance has not been reported in the literature. It was observed in the present study that excess-S produced more Met, while GSH content decreased under heat stress. On the contrary, the exogenous application of melatonin produced high GSH content, leading to a reduction in oxidative stress and lower ethylene evolution when compared to the excess-S treatment. Excess-S under non-stressed conditions also led to the approximately same level of GSH, and again, it could be interpreted that the Cys was diverted to ethylene via Met and not to GSH.

### 3.3. Melatonin with Excess-S Enhanced the Antioxidant Defense System to Reduce Oxidative Stress under High Temperature Stress

Melatonin is considered an endogenous free radical scavenger with the strongest antioxidant effect [12]. Due to high temperatures, an endogenous melatonin deficit caused oxidative stress in tomatoes [13]. Separate studies on melatonin and S have revealed that melatonin acts as an antioxidant, upregulates the activity of SOD, GR, and APX, and increases the gene expression of the enzymes involved in S uptake and assimilation, thereby increasing ascorbate and GSH content under heat stress [25,27]. In our study, the individual application of melatonin and excess-S improved the activity of antioxidant enzymes, but the application of melatonin in combination with excess-S to the mustard plant escalated SOD, APX, GR, and scavenging ROS activity, thus boosting stress tolerance under heat stress. Consistent with this, it was observed that melatonin, combined with S, alleviated La toxicity by strengthening the antioxidant system of the tomato plant [16]. Melatonin’s application to heat-exposed plants enhanced the antioxidant defense mechanism of tall fescue and positively reduced oxidative damage to improve physiological activities and growth [31].

Melatonin at 70 µM significantly increased the activities of the antioxidative enzymes CAT, GR, and GPX, which reduced heat-induced damaging effects in maize seedlings [32]. Individually, both melatonin and excess-S enhanced the antioxidative enzymes, and the combination proved to be the best because of efficient S-assimilation with melatonin under heat stress.

### 3.4. Melatonin and Excess-S under Heat Stress Regulate Ethylene Sensitivity for the Maximum Response

The negative impact of excess-S on plants’ photosynthesis and growth was due to excess ethylene evolution, which diminished photosynthesis and growth. Further, heat stress induced oxidative stress because of the accumulation of ROS [33]. Both ethylene and H_2_O_2_ acted together in a feedback loop in which ethylene induced H_2_O_2_ accumulation and H_2_O_2_ enhanced ethylene production, leading to the senescence of leaf and chlorosis under high temperature stress [34].

Melatonin in the presence of excess-S enhances GSH content while decreasing stress ethylene in heat-exposed plants. Stress ethylene’s reduction via the melatonin and excess-S combination resulted in the regulation of GSH levels, photosynthesis, and growth under heat stress even in a low-ATP-S cultivar. Ethylene sensitivity was, thus, enhanced via the melatonin treatment, which positively regulated photosynthesis and growth. S-assimilation plays a role in stress tolerance through the regulation of both ethylene and GSH under stress. In the present study, GSH acted as an antioxidant to remove ROS and changed to oxidized GSH (GSSG), which was again converted to GSH via GR, maintaining the GSH pool. Moreover, ethylene played an important role in the regulation of S-assimilation and was itself formed during the process of S-assimilation when the pool of Cys that formed was diverted to both GSH and Met and, from Met, moved to SAM and, finally, to ethylene [29]. In the case of excess-S, there was significantly the same level of GSH as under heat stress, although high Met content resulted in the formation of stress ethylene. Ethylene signaling decreased heat-induced oxidative stress and improved chlorophyll content in rice [35].

**Figure 9:**
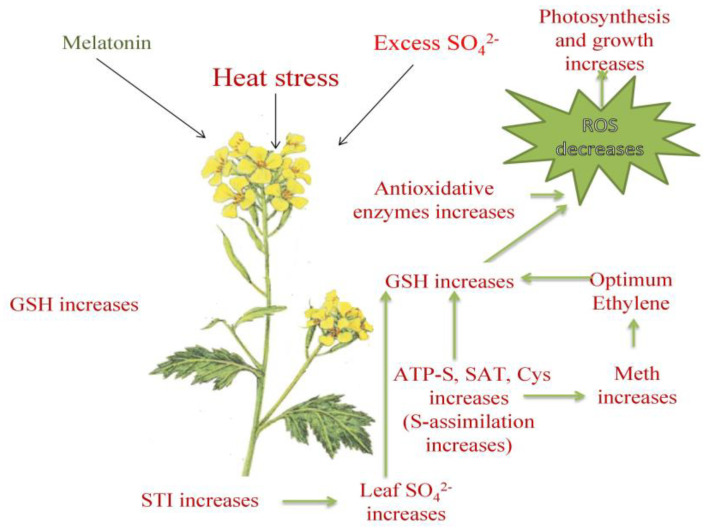
The mechanistic interaction between melatonin and ethylene that affects heat stress tolerance’s impact on excess-S availability.

In *Medicago* plants, melatonin treatment decreased ACS, the ethylene biosynthesis gene, and ERF, a transcription factor to protect plants from the effects of waterlogging [36]. Similarly, in this study, melatonin treatment, either individually or with excess-S, decreased stress ethylene evolution. The optimum ethylene concentration showed maximum responses that were achieved with the combined treatment of melatonin and excess-S. The stress ethylene that evolved with heat stress or excess-S was reduced due to the enhanced S-utilization and transport to form S-containing defense compounds, such as GSH, that scavenged ROS and reduced stress and ethylene. The inhibition of ethylene action through NBD confirmed the role of ethylene in melatonin and excess-S-mediated heat tolerance in SS2. NBD is an ethylene action inhibitor, and when it was applied to the plants, it resulted in the suppression of ethylene-mediated processes. Melatonin and S enhanced photosynthesis and growth via the regulation of ethylene action. We observed a decrease in the alleviation effect when NBD was supplemented. Melatonin helped in the utilization of excess-S, which contributed to optimum ethylene formation and higher GSH synthesis. This ethylene signaled stress-mediated responses because of an excess ethylene presence, and the negative effect of ethylene on photosynthesis and growth was observed. We obtained contrasting results to those reported in [37], wherein melatonin enhanced ethylene synthesis under salt stress via increased ACS activity [37]. Figure 9 depicts the mechanistic interaction between melatonin and ethylene that affects the availability of extra S during heat stress tolerance.

## 4. Material and Methods

### 4.1. Plant Material and Growth Conditions

Mustard (*Brassica juncea* L. Czern & Coss. var. SS2) seeds were disinfected with HgCl_2_ (0.01%), washed with double-distilled water, and sown in earthen pots (23-cm diameter) with acid-washed sand at the Department of Botany, Aligarh Muslim University, Aligarh, India. They were placed in an environmental growth chamber (Khera-Instruments, New Delhi, India) with a temperature set to 25 °C/14 °C, photosynthetically active radiation (PAR) of 660 µ mol m^−2^ s^−1^, and relative humidity of 62 ± 5%. Every other day, 150 mL of Hoagland’s nutrition solution (full-strength) was given to the plants in each pot. Following the emergence of seedlings (i.e., 10 days after sowing, DAS, with 4 leaf stage, 9.5 cm), the plants were subjected to high-temperature-stress treatment via keeping them at 40 °C for 6 h each day for 15 days, while all other growth conditions remained constant. The plants were allowed to recover at 25 °C (the optimal temperature) after 15 days of stress (9 leaf stage) and afterwards grown for the remainder of the experimental period (11 leaves, 18.2 cm in length). The control group of plants had the same optimum temperature (25 °C) throughout the 30-day experimental growth period. The SS2 cultivar showed low ATP-S activity and low STI, as reported in earlier studies [38], and, therefore, was included in this study.

To investigate the influence of melatonin, a hand sprayer was used to apply 100 µM of melatonin (25 mL per pot) to the foliage of both the high-temperature-stress-treated and control plants at 5 days after heat stress or 15 days after seedling emergence to ensure that the impact was entirely due to melatonin. The effect of melatonin treatment at 5 days following heat stress was evaluated 15 days later. Tween-20 (0.01%) was mixed in with both the melatonin and the control. Under the control and heat stress conditions, 2 mM of SO_4_^2−^ was given to the plants along with the growth media. The S was provided as MgSO_4_ and Mg^2+^ were maintained constantly at 2 mM in all treatments, including the control, via the addition of MgCl_2_.

To verify the role of ethylene in melatonin and excess-S mediated heat stress alleviation, an ethylene action inhibitor (100 µM of NBD) was supplied for the SO_4_^2−^ and melatonin treatment under heat stress. Four replicates were taken for each treatment, and all the parameters were evaluated at 30 DAS.

### 4.2. Measurement of Photosynthetic and Growth Parameters

In the fully expanded upper leaves, net photosynthesis (Pn), stomatal conductance (Gs), and intercellular CO_2_ concentration (Ci) were measured using an infrared gas analyzer (CID-340 Photosynthesis System, BioScience, Camas, WA, USA). The atmospheric CO_2_ content was 390 ± 5 µmol mol^−1^ during the experiment, and the photosynthetically active radiation (PAR) was 780 µmol m^−2^ s^−1^. The ratio of net photosynthesis to S content per unit leaf area was used to compute photosynthetic sulfur use efficiency (p-SUE). The activity was calculated after the addition of enzyme extract and 0.2 mM of ribulose-1,5-bisphosphate (RuBP). The method of Usuda [39] was adopted. The details of the procedure are included in Supplementary File S1.

The plants were carefully removed with their roots for growth measurements, adherent soil particles were removed, and blotting paper was used for drying. The plants were dried in an oven set to 80 °C until their weight became uniform. Finally, the plants were weighed to determine their dry mass. A leaf area meter (Systronics, LA211, New Delhi, India) was used to calculate the leaf area.

### 4.3. Measurement of S-Assimilation-Related Enzymes and Biomolecules

ATP-S activity was determined using the Lappartient and Touraine [20] method. SAT activity was measured via the adoption of Kredich and Tomkins’s [40] method. Cys, GSH, Met, and sulfate content was determined via the methods of Gaitonde [41], Griffith [42], Horn et al. [43], and Chesnin and Yang [44], respectively. Supplementary File S1 contains information on these methods.

### 4.4. Measurement of ACS Activity and Ethylene Evolution

The procedures for the assay of ACC synthase (EC 4.4.1.14) activity and ethylene evolution were adopted from Avni et al. [45] and Woeste et al. [46]. These procedures are outlined in the supplementary file.

### 4.5. Measurement of Oxidative Stress and Activity of Antioxidative Enzymes

The procedures provided by Okuda et al. [47] and Dhindsa et al. [48] to determine TBARS and H_2_O_2_ content, respectively, as well as the procedures to determine superoxide dismutase (SOD) activity by Giannopolitis and Ries [49] and Beyer and Fridovich [50], and the procedures to determine ascorbate peroxidase (APX) and glutathione reductase (GR) by Nakano and Asada [51] and Foyer and Halliwell [52], respectively, were used. These methods are described in Gautam et al. [7] and also presented in Supplementary File S1.

### 4.6. Statistical Analysis

SPSS 17.0 for Windows was used to perform statistical analysis of variance (ANOVA) on the data, which were then provided as treatments’ mean SEs (n = 4). For significant data at *p* < 0.05, the least significant difference (LSD) was determined. LSD test results showed that data following the same letter were not substantially different at *p* < 0.05. Origin Pro (v. 9.8) for Windows was used to perform principal component analysis and Pearson correlation.

## 5. Conclusions

Conclusively, melatonin, both alone and in the presence of excess-S, regulates photosynthesis in plants under heat stress by optimizing ethylene production. The presence of excess-S and melatonin meets the increased demand for S-containing compounds in heat stress tolerance by improving S-assimilation, STI, and GSH content while decreasing stress ethylene. The reduction in stress ethylene via melatonin and S combinations is the key reason for the enhanced photosynthesis and growth of heat-stress plants, which was validated by inhibiting ethylene action, resulting in a reduction in photosynthesis and growth even when melatonin and S were present. Under high temperature stress, there was a reduction in stress-induced ethylene via melatonin through two possibilities: firstly, via the reduction in ACS synthesis, and secondly, via the increase in GSH, which reduced oxidative stress and, thus, stress-induced ethylene. The results of this study could be exploited to develop stress tolerance, better photosynthesis, and better growth in cultivars that inherently have low ATP-S activity and low STI.

## Figures and Tables

**Table 1:** Content of hydrogen peroxide (H_2_O_2_) and thiobarbituric acid reactive substances (TBARS) and superoxide dismutase (SOD), ascorbate peroxidase (APX), and glutathione reductase (GR) activity of mustard (*Brassica juncea* L. cv. SS2) at 30 d after sowing. Plants were foliar treated with 100 µM of melatonin and/or 2 mM of SO_4_^2−^ (S) and grown with/without high temperature stress (HS; 40 °C for 6 h every day for 15 days). Data are presented as treatment means ± SEs (*n* = 4). Data followed by the same letter are not significantly different from the LSD test at *p* < 0.05. FW, fresh weight.

Treatments	H_2_O_2_ (nmol g^−1^ FW)	TBARS (nmol g^−1^ FW)	SOD (U mg^−1^ Protein min^−1^)	APX (U mg^−1^ Protein min^−1^)	GR (U mg^−1^ Protein min^−1^)
Control	21.8 ± 0.9 ^d^	08.0 ± 0.5 ^d^	6.73 ± 0.35 ^g^	2.03 ± 0.14 ^g^	0.14 ± 0.008 ^g^
HS	37.4 ± 1.7 ^a^	14.8 ± 1.2 ^a^	12.5 ± 0.58 ^f^	3.20 ± 0.19 ^f^	0.21 ± 0.014 ^ef^
S	26.6 ± 1.3 ^b^	9.88 ± 0.8 ^b^	13.9 ± 0.66 ^e^	2.70 ± 0.25 ^e^	0.28 ± 0.015 ^e^
Melatonin	11.4 ± 0.5 ^f^	3.66 ± 0.2 ^g^	14.7 ± 0.84 ^d^	4.50 ± 0.16 ^d^	0.34 ± 0.016 ^d^
HS + S	24.3 ± 1.3 ^c^	08.9 ± 0.5 ^cd^	17.8 ± 0.99 ^c^	7.09 ± 0.38 ^c^	0.39 ± 0.017 ^c^
Melatonin + HS	20.5 ± 1.2 ^d^	07.3 ± 0.2 ^de^	19.1 +1.15 ^b^	8.01 ± 0.4 ^b^	0.44 ± 0.018 ^b^
Melatonin + S + HS	15.3 ± 0.8 ^e^	4.94 ± 0.2 ^f^	22.6 ± 1.27 ^a^	9.4 ± 0.71 ^a^	0.52 ± 0.023 ^a^

**Table 2:** ATP-sulfurylase (ATP-S) and serine acetyl transferase (SAT) activity and content of cysteine (Cys), methionine (Meth), and reduced glutathione (GSH) of mustard (*Brassica juncea* L. cv. SS2) at 30 d after sowing. Plants were foliar treated with 100 µM of melatonin and/or 2 mM of SO_4_^2−^ (S) and grown with/without high temperature stress (HS; 40 °C for 6 h every day for 15 days). Data are presented as treatment means ± SEs (n = 4). Data followed by the same letter are not significantly different from the LSD test at *p* < 0.05. FW, fresh weight.

Treatments	ATPS (U mg^−1^ Protein min^−1^)	SAT (U mg^−1^ Protein min^−1^)	Cys (nmol g^−1^ FW)	Meth (nmol g^−1^ FW)	GSH (nmol g^−1^ FW)
Control	1.59 ± 0.79 ^g^	1.04 ± 0.39 ^g^	38.7 ± 1.3 ^g^	14.3 ± 0.5 ^f^	244 ± 13.7 ^f^
HS	1.73 ± 0.98 ^f^	1.28 ± 0.77 ^f^	43.2 ± 1.5 ^f^	48.2 ± 1.7 ^a^	287 ± 14.8 ^e^
S	1.91 ± 1.17 ^e^	1.44 ± 0.85 ^e^	48.9 ± 1.9 ^e^	34.7 ± 1.9 ^b^	292 ±15.9 ^e^
Melatonin	2.38 ± 1.46 ^b^	1.99 ± 0.98 ^b^	56.7 ± 2.3 ^d^	21.5 ± 2.1 ^e^	345 ± 19.6 ^c^
HS + S	2.09 ± 1.31 ^d^	1.69 ± 1.05 ^d^	49.2 ± 1.8 ^c^	22.11 ± 2.3 ^e^	318 ± 20.6 ^d^
Melatonin + HS	2.21 ± 1.38 ^c^	1.81 ± 1.29 ^c^	60.3 +2.5 ^b^	26.9 ± 2.4 ^d^	366 ± 22.4 ^b^
Melatonin + S + HS	2.74 ± 1.85 ^a^	2.26 ± 1.46 ^a^	69.5 ± 2.7 ^a^	30.4 ± 3.3 ^c^	455 ± 25.4 ^a^

**Table 3:** Root and leaf sulfate (SO_4_^2−^) content and sulfate transport index (STI) of mustard (*Brassica juncea* L. cv. SS2) at 30 d after sowing. Plants were foliar treated with 100 µM of melatonin and/or 2 mM of SO_4_^2−^ (S) and grown with/without high-temperature stress (HS; 40 °C for 6 h every day for 15 days). Data are presented as treatment means ± SEs (n = 4). Data followed by the same letter are not significantly different from the LSD test at *p* < 0.05. DW, fresh weight.

Treatments	Root SO_4_^2−^ Content (mg kg^−1^ DW)	Leaf SO_4_^2−^ Content (mg kg^−1^ DW)	STI (%)
Control	1.00 ± 0.02 ^e^	2.99 ± 0.28 ^e^	33.44 ± 5.44 ^f^
HS	0.74 ± 0.01 ^g^	2.05 ± 0.14 ^f^	36.10 ± 6.24 ^e^
S	0.88 ± 0.32 ^f^	3.08 ± 0.56 ^e^	28.57 ± 4.83 ^g^
Melatonin	1.54 ± 0.51 ^a^	3.68 ± 0.78 ^a^	41.85 ± 7.19 ^ab^
HS + S	1.23 ± 1.21 ^d^	3.17 ± 0.64 ^d^	38.8 ± 6.48 ^d^
Melatonin + HS	1.35 ± 1.46 ^c^	3.31 + 0.83 ^c^	40.78 ± 6.21 ^bc^
Melatonin + S + HS	1.44 ± 0.78 ^b^	3.43 ± 0.96 ^b^	41.98 ± 8.19 ^a^

**Table 4:** Net photosynthesis (*Pn*), stomatal conductance (*Gs*), and intercellular CO_2_ concentration (Ci) of mustard (*Brassica juncea* L. cv. SS2) at 30 d after sowing. Plants were foliar treated with 100 μM of melatonin and/or 2 mM of SO_4_^2−^ (S) and grown with/without high temperature stress (HS; 40 °C for 6 h every day for 15 days). Data are presented as treatment means ± SEs (n = 4). Data followed by the same letter are not significantly different from the LSD test at *p* < 0.05.

Treatments	Net Photosynthesis (µmol CO_2_ m^−2^ s^−1^)	Stomatal Conductance (mmol CO_2_ m^−2^ s^−1^)	Intercellular CO_2_ Concentration (µmol CO_2_ mol^−1^)
Control	11.1 ± 0.78 ^d^	342 ± 14.2 ^b^	193 ± 10.11 ^c^
HS	6.9 ± 0.22 ^g^	245 ± 13.4 ^e^	141 ± 6.99 ^f^
S	8.3 ± 0.66 ^f^	289. ± 12.3 ^d^	152 ± 13.68 ^e^
Melatonin	15.7 ± 0.83 ^a^	429 ± 10.4 ^a^	278 ± 10.67 ^a^
HS + S	10.3 ± 0.71 ^e^	310 ± 13.8 ^c^	156 ± 14.4 ^d^
Melatonin + HS	13.4 ± 0.62 ^c^	329 ± 17.1 ^b^	200 + 11.12 ^c^
Melatonin + S + HS	14.4 ± 0.81 ^ab^	418 ± 19.6 ^a^	261 ± 12.6 ^ab^

## Data Availability

Data are contained within the article and supplementary material.

## Supplementary Materials

The following supporting information can be downloaded at https://www.mdpi.com/article/10.3390/plants12173160/s1, S1: the detailed methodology.
