# Supplementary material for: Melatonin Reverses High-Temperature-Stress-Inhibited Photosynthesis in the Presence of Excess Sulfur by Modulating Ethylene Sensitivity in Mustard
Source: Plants (Basel). 2023 Sep 2;12(17):3160. doi: 10.3390/plants12173160 (PMC10490298; doi:10.3390/plants12173160)
Supplement: Supplementary file 1 [file plants-12-03160-s001.zip › Supplementary file S1.pdf]

Melatonin Reverses High-Temperature-Stress-Inhibited Photosynthesis in the Presence of Excess Sulfur by Modulating Ethylene Sensitivity in Mustard

Noushina Iqbal [1], Zebus Sehar [2], Mehar Fatma [2], Sheen Khan [2], Ameena Fatima Alvi [2], Iqbal R. Mir [2], Asim Masood [2] and Nafees A. Khan [2,*]

[1] Department of Botany, Jamia Hamdard, New Delhi 110062, India

[2] Plant Physiology and Biochemistry Laboratory, Department of Botany, Aligarh Muslim University, Aligarh 202002, India

* Correspondence: naf9.amu@gmail.com

Materials and methods

1.1 Determination of Rubisco Activity

Ribulose-1,5-bisphosphate carboxylase/oxygenase (Rubisco) activity was measured using Usuda (1985) method. Leaf tissue (1.0 g) were homogenized in a chilled mortar and pestle with ice-cold extraction buffer containing 0.25 M Tris-HCl (pH 7.8), 0.0025 mM EDTA, 0.05 mM $MgCl_2$, and 37.5 mg DTT for enzyme extraction. The homogenate was centrifuged at 10,000× g for 10 min at 4 °C. The supernatant obtained after centrifugation was used to assay the enzyme. The reaction mixture contained 100 mM Tris-HCl (pH8.0), 10 mM $MgCl_2$, 40 mM $NaHCO_3$, 4.0 mM ATP, 0.2 mM NADH, 5.0 mM DTT, 0.2 mM EDTA, 1.0 U of glyceraldehydes-3-phosphodehydrogenase and 1.0 U of 3-phosphoglycerate-kinase and 0.2 mM of ribulose1,5-bisphosphate (RuBP).

1.2 Determination of S-assimilation enzymes and biomolecules

ATP-S activity was determined using Lappartient and Touraine (1996) method. One g fresh leaf tissue was ground at 4 ºC in a buffer consisting of 10 mM Na2EDTA, 20 mM Tris-HCl (pH 8.0), 2 mM dithio-threitol (DTT), and 0.01 g per cm3 polyvinylpyrrolidone (PVP), using 1 : 4 (m/v) tissue to buffer ratio. The homogenate was centrifuged at 20 000×g for 10 min at 4 ºC. The supernatant was used for in vitro ATP-sulfurylase assay. The enzyme activity was measured using molybdate-dependent formation of pyrophosphate. The reaction was initiated by adding 0.1 cm^3 of extract to 0.5 cm^3 of the reaction mixture, which contained 7 mM $MgCl_2$, 5 mM of Na_2MoO_4, 2 mM of Na_2ATP, and 0.032 units per cm^3 of sulfate-free inorganic pyro-phosphate in 80 mM Tris-HCl buffer (pH 8.0). Another aliquot from the same extract was added to the same reaction mixture except that Na_2MoO_4 was absent. Incubations were carried out at 37 ºC for 15 min, after which phosphate was determined spectrophotometrically.

SAT activity was measured by adopting Kredich and Tomkins (1966) method. In an ice-cold extraction buffer, fresh leaf tissues (500 mg) were ground. The buffer contained Tris–HCl at 8.0 pH and 100 mM concentration, KCl (100 mM), $MgCl_2$ (20 mM), Tween 80 (1%), and DTT (10 mM). The total volume of extraction buffer was 2 ml. After grounding, centrifugation was done at 11,600g at 4 ˚C for 10 min and the supernatant was utilized for SAT essay. The reaction mixture contained acetyl CoA (0.1 mM), Tris–HCl (50 mM) at pH 7.6, DTNB (1 mM), 1 mM EDTA, and 1 mM l-serine in 1 ml. At 25 ˚C when enzyme were added, the initial activity was calculated by recording the absorbance increase at 412 nm and the rates were estimated utilizing the extinction coefficient of 13,600 for thionitrobenzoic

acid. A blank excluding l-serine but having the rest of the material was run simultaneously, and the reading was subtracted from the reaction rate that is obtained with l-serine.

The Giatonde (1967) method was utilized for the estimation of Cys content in leaves. Fresh leaf (500 mg) was homogenized in 5% (w:v) ice-cold perchloric acid. The suspension was centrifuged at 2,800 g for 1 h at 5°C and supernatant was filtered. After that, 1 mL of filtrate was treated with acid ninhydrin reagent and the absorption was read at 580 nm. The amount of Cys was calculated using the calibration curve obtained for standard Cys.

The Griffith (1980) method was utilized for the estimation of GSH content, respectively. Reduced glutathione was assayed by an enzyme recycling procedure in which it was sequentially oxidized by 5, 5-dithiobis-2-nitrobenzoic acid (DTNB) and reduced by NADPH in the presence of GR. For specific assay of GSSG, GSH was masked by derivatization with 2-vinylpyridine. Fresh leaf tissues (500 mg) were ground in liquid nitrogen using mortar and pestle and suspended in 2 ml of 5% (w:v) sulfosalicylic acid. The centrifugation was done at 12,000×g for 10 min. A 300 μL aliquot of the supernatant was removed and neutralized by the addition of 18 μL 7.5 M triethanolamine. To determine concentrations of GSH plus GSSG, one 150 μL sample was then used. Another sample was pre-treated with 3 μL 2-vinylpyridine for 60 min at 20ºC to mask the GSH by derivatization, to allow the subsequent determination of GSSG alone. In each case, 50 μL aliquots of the two types of samples were mixed with 700 μL 0.3 mM NADPH, 100 μL DTNB, and 150 μl buffer containing 125 mM sodium phosphate, 6.3 mM EDTA (pH 6.5). A10 μL aliquot of GR (5 U ml^{-1}) was then added and the change in absorbance at 412 nm was monitored at 30ºC. The standard curve was prepared from GSH, covering a range of 5-55 nmol. For oxidized glutathione, a standard curve covering a range of 1-5 nmol was used.

The content of methionine was determined by adopting Horn et al. (1946) method. Fresh leaf samples measuring 500 mg were taken and mixed with 20.0 mL of HCl (6.0 N) for 20-24 h. They were then evaporated in a water bath, and activated charcoal (1.0 g) was added to it. The filtrate was collected to which de-ionized water (4.0 mL) plus 5 N-NaOH (2.0 mL) and sodium nitroprusside (0.1 mL) plus glycine solution (3%, 2 mL) was added. At last, phosphoric acid (4.0 mL) was added, and the intensity of colour was read at 450 nm.

For the determination of sulfate content, 100 mg of fine dried powder of leaf and root were digested in mixture of concentrated HNO_3 and 60% $HClO_4$ (85:1 v/v) using Chesnin and Yien (1950) turbidimetric method. STI was calculated by the ratio of root sulfate to leaf sulfate content and expressed as %.

1.3 Determination of ACS activity and ethylene evolution

ACC synthase (EC 4.4.1.14) activity was determined by the methods of Avni et al. (1994) and Woeste et al. (1999). The leaf tissues were grounded in N-[2-hydroxyethyl piperazine-N′ -2-ethane sulfonic acid] (HEPES) buffer having pH of 8.0 and 100 mM strength, containing dithiothreitol (DTT, 4 mM), pyridoxal phosphate (2.5 mM) and polyvinylpyrrolidone (25%). After homogenization, it was centrifuged for 15 min at 12 000 g. To 1 ml of supernatant in a tube, 0.1 ml AdoMet (5 mM) was added and was incubated at 22 °C for 2 h. The ACC formed was determined through its conversion to ethylene when 0.1 ml $HgCl_2$ (20 mM) was added with 0.1 ml of a 1:1 mixture of saturated ice cold NaOH/NaCl. The control set was devoid of AdoMet. For ethylene evolution, leaf material was trimmed into small pieces and then transferred to tubes which were placed for 2h in light.

Ethylene was measured by cutting 0.5 g of leaf material into small pieces that were placed into 30 mL tubes containing moist paper to minimize evaporation from the tissue and were stoppered with secure rubber caps and placed in light for 2 h under the same condition used for plant growth. An earlier experiment showed that 2 h incubation time was adequate for ethylene detection without the interference of wound-induced ethylene, which began after 2 h of leaf incubation. A 1 mL gas sample

from the tubes was withdrawn with a hypodermic syringe and assayed on a gas chromatograph (Nucon 5700, New Delhi, India) equipped with a 1.8 m Porapack N (80–100 mesh) column, a flame ionization detector and data station. Nitrogen was used as the carrier gas. The flow rates of nitrogen, hydrogen and oxygen were 30, 30, and 300 mL min^{-1}, respectively. The detector was set at 150°C. Ethylene was identified based on the retention time and quantified by comparison with peaks from standard ethylene concentration

1.4 Measurement of oxidative stress

Determination of H_2O_2 and TBARS Content

To estimate H_2O_2, the method of Okuda et al. (1991) was utilized and Dhindsa et al. (1981) method was used for TBARS content. 500 mg of fresh leaf tissue was homogenized in ice-cold 200 mM perchloric acid ($HClO_4$), followed by centrifugation at 1200× g for 10 min. Next, the supernatant was neutralized with 4 M KOH. The homogenate was further centrifuged at 500× g for 3 min for the removal of insoluble potassium perchlorate. For the determination of H_2O_2, the reaction mixture (1.5 mL) contained 1 mL of the eluate, 80 µL of 3-methyl-2-benzothiazoline hydrazone, 400 µL of 12.5 mM 3-(dimethylamino) benzoic acid in 0.375 M phosphate buffer (pH 6.5), and 20 µL of peroxidase (0.25 unit). The reaction was initiated with the addition of peroxidase at 25 °C, and the resulting increase in absorbance was estimated at 590 nm on a spectrophotometer.

For TBARS, fresh leaf samples (500 mg) were ground in 0.25% 2-thiobarbituric acid (TBA) in 10% trichloroacetic acid (TCA) using a mortar and pestle. The mixture was heated at 95 °C for 30 min, rapidly cooled in an ice bath, and centrifuged at 10,000× g for 10 min. To 1 mL of the resulting supernatant, 4.0 mL of 20% TCA containing 5% TBA was added. The absorbance of the supernatant was read at 532 nm and corrected for non-specific turbidity by subtracting the absorbance of the same at 600 nm. TBARS content was calculated using an extinction coefficient of 155 mM^{-1} cm^{-1}.

1.5 Determination of Antioxidant Enzymes Activity

Superoxide dismutase (SOD) was measured by adopting Giannopolitis and Ries (1977) and Beyer and Fridovich (1987) methods. Ascorbate peroxidase activity (APX; EC, 1.11.1.11) was measured following the method of Nakano and Asada (1981)

Superoxide Dismutase (SOD)

To 5.0 mL of reaction mixture containing 5 mM HEPES (pH 7.6), 0.1 mM EDTA, 50 mM Na_2CO_3 (pH 10.0), 13 mM methionine, 0.025% (v/v) Triton X-100, 63 μmol NBT, and 1.3 μmol riboflavin was mixed with the enzyme-containing extract. The reactants were then placed in bright light (360 μmol m^{-2} s^{-1}) for 15 min, while a corresponding control was not illuminated to allow for the correction of background absorbance. One unit of SOD is defined as the amount of enzyme needed to inhibit NBT reduction by 50% (as measured by the absorbance at 560 nm).

Ascorbate Peroxidase (APX)

The assay mixture (1.0 mL) contained phosphate buffer (50 mM, pH 7.0), 0.1 mM EDTA, 0.5 mM ascorbate, 0.1 mM H_2O_2, and enzyme extract, and was observed at 290 nm for 1 min using a spectrophotometer. A decrease in absorbance was observed as soon as the reaction was started (i.e., upon the addition of H_2O_2). An extinction coefficient of 2.8 mM^{-1} cm^{-1} was used when computing APX activity. One unit of APX is defined as the amount necessary to decompose one μmol of substrate per min at 25 °C.

Glutathione Reductase (GR)

The method of Foyer and Halliwell (1976) was adopted for GR The reaction mixture (3.0 mL) contained phosphate buffer (25 mM, pH 7.8), 0.5 mM GSSG, 0.2 mM NADPH, and the enzyme extract. The reaction was initiated upon the addition of GSSG, and a decreasing trend in absorbance was immediately evident. An extinction coefficient of 6.2 mM^{-1} cm^{-1} was used when quantifying GR activity. One unit of enzyme is defined as the amount necessary to decompose one μmol of NADPH per min at 25 °C.

References

1. Usuda, H. The activation state of ribulose 1, 5-bisphosphate carboxylase in maize leaves in dark and light. *Plant Cell Physiol*. **1985,** *26*, 1455-1463.

2. Lappartient, A.G.; Touraine, B. Demand-driven control of root ATP sulfurylase activity and $SO4^{2-}$ uptake in intact canola (the role of phloem-translocated glutathione). *Plant Physiol.* **1996**,*111*, 147-157.

3. Kredich, N.M.; Tomkins, G.M. The enzymic synthesis of L-cysteine in Escherichia coli and Salmonella typhimurium. *J. Biol. Chem*. **1966**, *241*, 4955-65.

4. Gaitonde, M.K. A spectrophotometric method for the direct determination of cysteine in the presence of other naturally occurring amino acids. *Biochem. J.* **1967**, *104*, 627.

5. Griffith, O.W. Determination of Glutathione and Glutathione Disulfide Using Glutathione Reductase and 2-Vinylpyridine. *Anal. Biochem*. **1980,** *106,* 207-212.
6. Horn, M.J.; Jones, D.B.; and Blum, A.E. Colorimetric determination of methionine in proteins and foods. *J. Biol. Chem*. **1946**, *166*, 313-320.

7. Chesnin, L.; Yien, C.H. Turbidimetric Determination of Available Sulphates. *Soil Sci. Soc. America J.* **1950**, *15*, 149-151.

8. Avni, A.; Bailey, B.A.; Mattoo, A.K.; Anderson, J.D. Induction of ethylene biosynthesis in *Nicotiana tabacum* by a *Trichoderma viride* xylanase is correlated to the accumulation of 1-aminocyclopropane-1-carboxylic acid (ACC) synthase and ACC oxidase transcripts. *Plant Physiol.* **1994**, *106*, 1049-1055.

9. Woeste, K.E.; Ye, C.; Kieber, J.J. Two Arabidopsis mutants that overproduce ethylene are affected in the posttranscriptional regulation of 1-aminocyclopropane-1-carboxylic acid synthase. *Plant Physiol.* **1999**, *119*, 521-530.

10. Okuda, T.; Matsuda, Y.; Yamanaka, A.; Sagisaka, S. Abrupt increase in the level of hydrogen peroxide in leaves of winter wheat is caused by cold treatment. *Plant Physiol*. **1991**, *97*, 1265-1267.
11. Dhindsa, R.S.; Plumb-Dhindsa, P.A.M.E.L.A.; Thorpe, T.A.; Leaf senescence: correlated with increased levels of membrane permeability and lipid peroxidation, and decreased levels of superoxide dismutase and catalase. *J. Exp. Bot.* **1981**, *32,* 93-101.
12. Giannopolitis, C.N.; Ries, S.K. Superoxide dismutases: I. Occurrence in higher plants. *Plant Physiol.* **1977**, *59*, 309-314.
13. Beyer Jr, W.F.; Fridovich, I. Assaying for superoxide dismutase activity: some large consequences of minor changes in conditions. *Anal. Biochem.* **1987**, *161*, 559-566.
14. Nakano, Y.; Asada, K. Hydrogen peroxide is scavenged by ascorbate-specific peroxidase in spinach chloroplasts. *Plant Cell Physiol.* **1981**, *22*, 867-880.
15. Foyer, C.H.; Halliwell, B. The Presence of Glutathione and Glutathione Reductase in Chloroplasts: A Proposed Role in Ascorbic Acid Metabolism. *Planta* **1976**, *133*, 21-25.
